# Ubiquitinated Hepatitis D Antigen-Loaded Microvesicles Induce a Potent Specific Cellular Immune Response to Inhibit HDV Replication in Vivo

**DOI:** 10.1128/Spectrum.01024-21

**Published:** 2021-12-15

**Authors:** Ting Yao, Mengjiao Lv, Siyuan Ma, Jinmei Chen, Yi Zhang, Yongsheng Yu, Guoqing Zang, Xiaohua Chen

**Affiliations:** a Department of Infectious Diseases, Shanghai Jiao Tong University Affiliated Sixth People’s Hospital, Shanghai, China; University of Florida

**Keywords:** HDV, dendritic cell-derived exosomes, ubiquitination, specific immune response, JAK/STAT pathway, hepatitis, exosome

## Abstract

Hepatitis D is the most severe form of human viral hepatitis and currently lacks an efficient therapy. Dendritic cell-derived exosomes (Dexs) have been found to induce immune responses capable of eliminating viruses. However, the therapeutic potential of antigen-loaded exosomes in hepatitis D is still unknown. Recently, we designed exosomes loaded with ubiquitinated hepatitis delta virus (HDV) small delta antigen (Ub-S-HDAg) and then treated mice bearing replicating HDV with these exosomes to explore their antiviral effect and mechanism. Mature dendritic cell-derived exosomes (mDexs) were loaded with Ub-S-HDAg and their antivirus function was evaluated in mice with HDV viremia. Furthermore, the proportion of CD8^+^ cells, the ratio of Th1/Th2 cells, the postimmunization levels of cytokines were explored, and the Janus kinases (JAK)/signal transducer and activator of transcription (STAT) pathway was evaluated with a JAK2 inhibitor AG490. In Ub-S-HDAg-Dexs group, the HDV RNA viral load was significantly decreased compared with other groups by CD8^+^ cell enrichment and an increase Th1/Th2 cell ratio. Furthermore, lymphocyte infiltration was increased, while the HDAg level was decreased in mouse liver tissue. However, there were no significant differences in HBV surface antigen (HBsAg), alanine aminotransferase (ALT), or aspartate aminotransferase (AST) levels among the groups. Moreover, p-JAK2, p-STAT1, p-STAT4, STAT1, and STAT4 expression was increased in Ub-S-HDAg-Dexs group. In conclusion, Ub-S-HDAg-Dexs might be a potential immunotherapeutic agent for eradicating HDV by inducing specific cellular immune response via the JAK/STAT pathway.

**IMPORTANCE** Hepatitis D is the most severe viral hepatitis with accelerating the process of liver cirrhosis and increasing the risk of hepatocellular carcinoma. However, there are no effective antiviral drugs. Exosomes derived from mature dendritic cells are used not only as immunomodulators, but also as biological carriers to deliver antigens to induce robust immune response. Based on these properties, exosomes could be used as a biological immunotherapy by enhancing adaptive immune response to inhibit hepatitis D virus replication. Our research may provide a new therapeutic strategy to eradicate HDV in the future.

## INTRODUCTION

Hepatitis delta virus (HDV) infection causes serious hepatitis with rapid disease progression to cirrhosis and hepatocellular carcinoma and leads to severe acute hepatitis in 50% to 70% of people with superinfection, which occurs only in individuals with hepatitis B virus (HBV) infection (1). Of the 350 million chronic HBV carriers worldwide, more than 15 million are coinfected with HDV (2, 3). However, the rate may be higher in several areas, such as Mongolia. It has been revealed that approximately 60% of the Mongolian HBV surface antigen (HBsAg) positive population is coinfected with HDV (4). Nevertheless, the current treatment option is interferon alpha (IFN-α), which is effective in only a minority of patients. This therapy has a low virological response rate (<30%) at 24 weeks posttreatment and a high late relapse rate (>50%) with obvious side effects and a high cost (3). Bulevirtide has been approved for the treatment of chronic HDV infection. However, the optimal treatment duration is unknown. Treatment should be continued as long as a clinical benefit is evident (5). HDV-specific T cell proliferation and cytokine production are weak, and persistent viral infection is often characterized by the presence of hyporesponsive antiviral T cells that are unable to control the infection (6, 7).

Exosomes are released by almost all kinds of cell types and constitute a subset of extracellular vesicles (EVs) that are 30 to 1150 nm in diameter. Exosomes have been reported to contain nucleic acids, proteins, lipids, and other bioactive substances. When exosomes are taken up by other cells, these cargoes are safely transferred to influence the phenotype of the recipient cells and participate in regulatory processes, such as those influencing tissue repair, tumor initiation and progression, and immunity (8). Dendritic cells (DCs) are professional antigen-presenting cells (APCs) that can modulate T cell immunity (9). Exosomes from APCs carry major histocompatibility complex (MHC) class I (MHC I) and T cell costimulatory molecules on their surface, suggesting that they play important roles in immune regulation (10). To date, exosomes have been studied as drug delivery vehicles, vaccine and immunotherapy tools, and carriers for biomarkers. Previous reports have demonstrated that an exosome-bound antigen can induce a more potent antigen-specific antitumor or antiviral immune response than the soluble form of the antigen (11, 12).

Janus kinases (JAKs) compose a family of cytosolic protein kinases including four members (JAK1, JAK2, JAK3, and tyrosine kinase 2 [TYK2]) that are crucially involved in immune signaling (13). Signal transducer and activator of transcription (STAT) proteins compose a family of factors implicated in a variety of biological processes (14). The JAK/STAT pathway plays an important role in inflammatory processes. Upon a cytokine-induced conformational change in the corresponding receptor, JAKs are activated by transphosphorylation and subsequently phosphorylate the intracellular receptor domains. The latter recruit STAT proteins, which are phosphorylated by JAKs, undergo dimerization and translocate to the nucleus, where they act as transcription factors (13, 15). Previous studies have illustrated that activating specific immune responses via the JAK/STAT pathway can inhibit viral replication (15, 16). AG490, as a JAK2 inhibitor, was previously shown to inhibit the JAK/STAT signaling pathway (17).

Ubiquitin (Ub) is an evolutionarily conserved protein that posttranslationally marks proteins for degradation (18). Ubiquitination is crucial in immunity and regulates pattern recognition receptor signaling, which mediates both the innate immune response and DCs maturation (18, 19). Our previous work has shown that lentiviral vectors encoding ubiquitinated HBV core antigen (Ub-HBcAg) can promote DCs maturation, enhance Th1-type immune responses, and induce HBcAg-specific cytotoxic T lymphocytes (CTLs) *in vivo* (20).

In the present study, we explored whether engineered exosomes enhance the virus-specific immune response to inhibit HDV replication. Lysosome-associated membrane glycoprotein 2b (Lamp2b) can localize HDV antigens on the cell membrane and selectively enrich antigenic peptides in exosomes (21, 22). We constructed a recombinant Ub-S-HDV small delta antigen (HDAg)-Lamp2b plasmid and transfected it into bone marrow-derived DCs to produce exosomes. The specific cellular immune response to inhibit HDV replication was induced by Ub-S-HDAg-containing exosomes derived from mature DCs (Ub-S-HDAg-Dexs) *in vivo*. We demonstrated the CD8^+^ cell enrichment and an increased Th1/Th2 cell ratio contributed to the virus clearance and this effect attributed to activated JAK/STAT pathway.

## RESULTS

### Isolation, characterization, and labeling of Ub-S-HDAg-Dexs.

**FIG 1:**
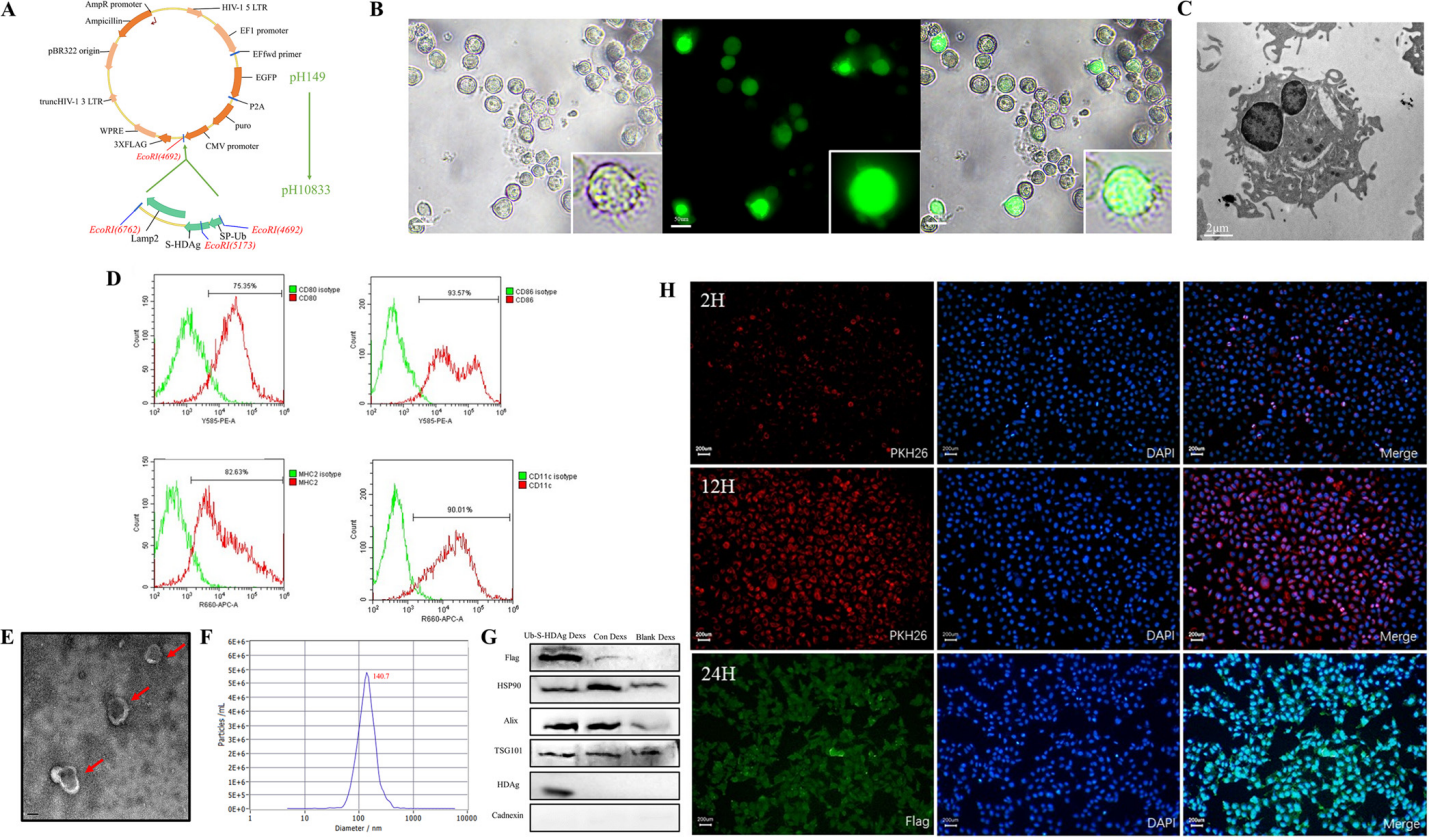
Isolation and characterization of Ub-S-HDAg-Dexs. (A) The structure of the recombinant plasmid. (B) After transfection with the lentivirus, mDCs were obtained by LPS stimulation for 24 h, and the typical mDC morphology was observed under light and fluorescence microscopes. The mDCs expressed the green fluorescence. Bars, 50 μm. (C) The typical mDC morphology was observed under transmission electron microscopes. Bars, 2 μm. (D) mDCs were stained with anti-CD11C, anti-CD86, anti-CD80, and anti-MHC II antibodies or isotype-matched control antibodies and analyzed by flow cytometry. (E) The Ub-S-HDAg-Dex ultrastructure determined by transmission electron microscopy (red arrows). Bar, 100 nm. (F) The size distribution profile of mDexs determined using the Malvern NanoSight NS300 system, showing a size peak of 140.7 nm. (G) Expression of the positive exosomal markers Alix, Hsp90, and Tg101 as well as that of the Flag-tagged protein and HDAg was detected in Dex lysates, and the negative marker calnexin was not detected by Western blotting. (H) DC2.4 cells were incubated with PKH26-labeled mDexs (red) for 6h, 12h, 24h, and stained with an anti-Flag antibody and DAPI for fluorescence imaging.

To generate Ub-S-HDAg-loaded exosomes, we fused the Ub-S-HDAg gene with the N-terminus of the murine Lamp2b protein to form the pLV-EGFP-Ub-S-HDAg-Lamp2b-3Flag plasmid (Fig. 1A) and the sequence was detected to compare the predicted sequence of SP-Ub-S-HDAg-Lamp2b (Fig. S1), which was transfected into imDCs. High fluorescence was observed, and the rate of positivity reached up to 60% to 70% at 72 h (Fig. S2). As shown in Fig. 1B and C, after LPS stimulation, suspension cell clusters were found to increase, and a typical morphology, such as branch-like protrusions and aggregation of branching structures among cells, appeared. In addition, cells showed positive staining for CD11c, CD80, MHC-II, and CD86 at rates of 90.01%, 75.35%, 82.63%, and 93.57%, respectively (Fig. 1D), indicating maturation of the DCs.

We extracted and purified exosomes from the culture supernatants by ultracentrifugation and ultrafiltration. Typical exosomal structures were observed by transmission electron microscopy (TEM) (Fig. 1E). Nanoparticle tracking analysis (NTA) showed that the extracts had a narrow size distribution, with a mean particle diameter of 104.7 nm (Fig. 1F). These data were consistent with those previously reported for typical exosomes (23). The positive exosomal markers Alix, Hsp90, and TG101 and negative marker Calnexin indicated that the extracts comprised exosomes (Fig. 1G). Positivity for the Flag-tagged protein and HDAg indicated that DCs were transfected successfully and expressed S-HDAg. Additionally, S-HDAg was present in isolated exosomes.

To confirm these mDexs could be taken up by cells, mDexs were incubated with DC2.4 cells. Over time, it became increasingly obvious that DC2.4 cells were positive for the dye PKH26 used to label the exosomes. This finding indicated that exosomes could be taken up by DC2.4 cells. Because the Flag protein was derived from only exosomes produced by successfully transfected DCs, positivity for Flag in DC2.4 cells, combined with the results obtained by Western blotting (Fig. 1H), indicated that exosomes carrying the S-HDAg protein entered these cells.

### Establishment of intrahepatic HDV replication in HBV transgenic mice.

**FIG 2:**
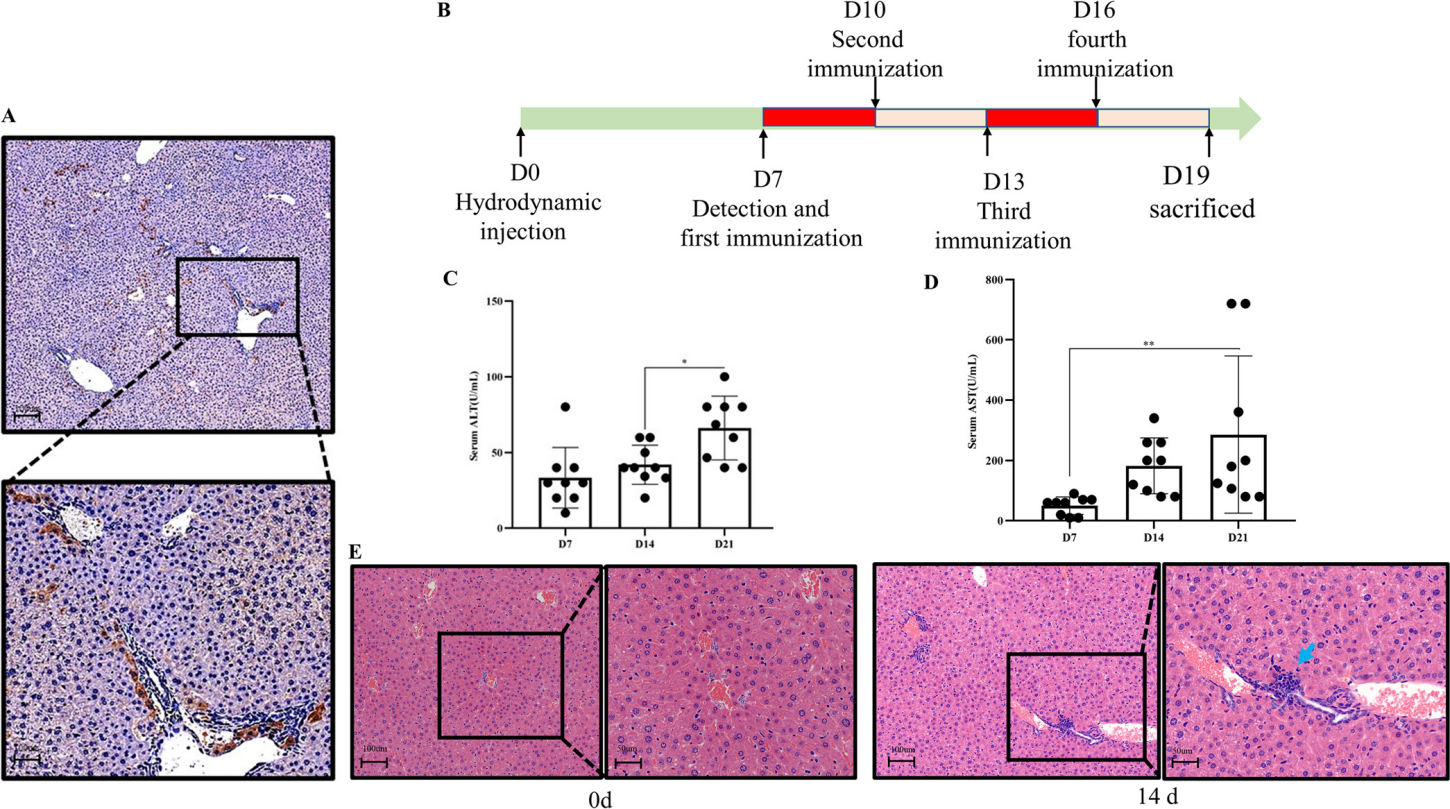
Establishment of intrahepatic HDV replication in HBV transgenic mice. (A) The positive immunohistochemical staining pattern of HDAg in HBV transgenic mice at 7 days after hydrodynamic injection. (B) Schematic of the HDV replication model and treatment protocol. On the seventh day after HDV-encoding plasmid pCMV-HDV-I (+) being injected, the mice were injected subcutaneously Dexs in the hind footpads every 3 days for a total of four times. (C, D) Changes in ALT and AST levels in the serum at 7, 14, and 21 days after hydrodynamic injection. (E) Liver sections obtained at 0 and 14 days were analyzed by H&E staining. Piecemeal necrosis (blue arrow), hepatocyte hypertrophy, and sandglass hepatocytes. Data are presented as mean ± SD. Significance was calculated by one-way ANOVA tests: *, *P* = 0.05; **, *P* = 0.01.

We established a mouse model of HDV replication to evaluate the potential efficacy of mDexs *in vivo*. According to a previous process, we delivered the HDV-encoding plasmid pCMV-HDV-I (+) into HBV transgenic mice by hydrodynamic transfection (24). As shown in Fig. 2A, 20% to 25% of the hepatocytes in the mouse displayed brown staining by immunohistochemical analysis of liver sections, which indicated hepatocytes were positively stained for HDAg and supported HDV replication. For viral load determination, we constructed a standard curve (Fig. S3) using the purified plasmid pT7GM to calculate the HDV load in the serum by qRT–PCR analysis. As shown in Table 1, the viral load reached approximately 10^6^ copies/mL at day 7. As shown in Fig. 2C and D, as HDV replicated, the levels of alanine aminotransferase (ALT) and Aspartate aminotransferase (AST) increased. Pathological analysis revealed diffuse hepatocyte hypertrophy, inflammatory cell infiltration, piecemeal necrosis, and focal/multifocal single-cell necrosis in liver of HDV replicated mice while the HBV transgenic mice had no liver injury (Fig. 2E). (25).

**TABLE 1:** Serum HDV RNA log (copies/mL) at day 7

Mouse no.	Log (copies/mL)	Mouse no.	Log (copies/mL)	Mouse no.	Log (copies/mL)
1	6.77	11	5.21	21	6.17
2	6.74	12	7.24	22	6.28
3	6.74	13	5.00	23	5.79
4	4.78	14	5.01	24	6.38
5	6.61	15	7.57	25	5.83
6	4.84	16	7.83	26	6.74
7	6.06	17	7.44	27	5.93
8	5.57	18	5.85	28	6.33
9	5.01	19	4.12	29	6.39
10	5.51	20	5.13	30	6.82

### Exosomes mediate antiviral effects via an activated immune response.

**FIG 3:**
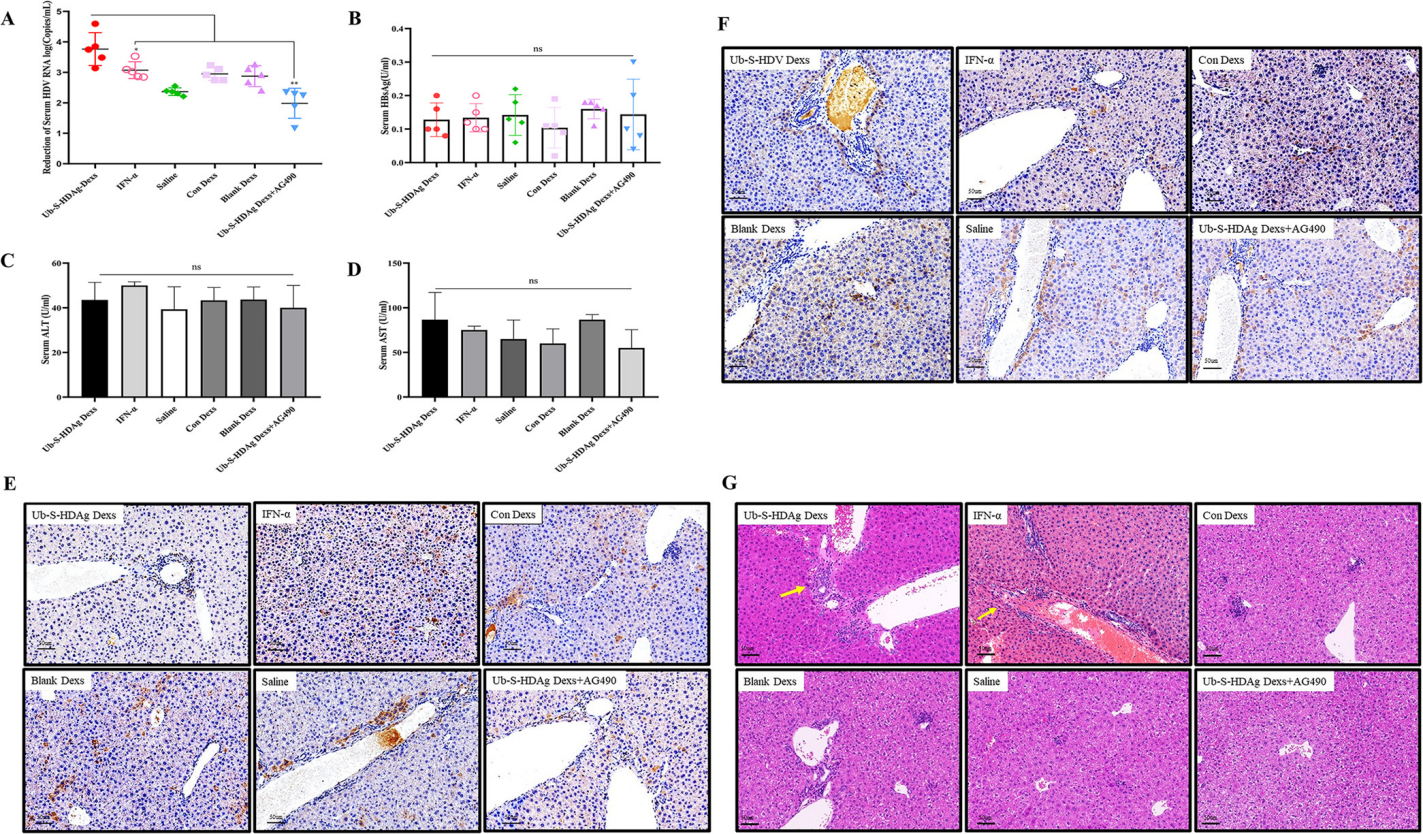
Ub-S-HDAg-Dexs have antiviral effects mediated by the activation of a specific immune response. (A) Mice hydrodynamically transfected to produce HDV viremia were treated with different Dexs for 12 days prior to sacrifice. Serum HDV RNA was quantitated by RT-PCR. (B, C, and D) The levels of HBsAg, ALT, and AST in the serum in each group after immunotherapy. (E and F) Immunohistochemical staining for HBsAg and HDAg in liver sections from each group after immunotherapy. Brown-red spots indicate the characteristic nuclear staining pattern of HBsAg and HDAg. Bars, 50 μm. (G) Liver sections were analyzed by H&E staining after immunotherapy. Inflammatory infiltrates (yellow arrow) were observed in the Ub-S-HDAg-Dex group, and hypertrophy and sandglass hepatocytes were obvious in all groups except the Ub-S-HDAg-Dex and IFN-α groups by light microscopy. Bars, 50 μm. Significance was calculated by one-way ANOVA tests: *, *P* = 0.05; **, *P* = 0.01; ns, nonsignificant.

To verify whether modified mDexs could activate T cells to clear HDV in mice, we randomly divided the HDV replication model mice into six groups: Ub-S-HDAg-Dexs, Con-Dexs, Blank Dexs, Ub-S-HDAg-Dexs+AG490, IFN-α (positive control), and saline (blank control). We treated mice with exosomes via the footpad every 3 days for a total of 4 times. The mice were sacrificed on day 12 post treatment to analyze the serum HDV RNA load and biochemical indicators. The process is shown in Fig. 2B. As shown in Fig. 3A, the HDV viral load in the Ub-S-HDAg-Dexs group was significantly reduced compared with that in the other groups. As expected, IFN-α inhibited HDV replication, but the antiviral effect of Ub-S-HDAg-Dexs was stronger than that of IFN-α (*P <* 0.05). The levels of HBsAg (Fig. 3B), ALT, and AST (Fig. 3C and D) in the serum were not significantly different among the groups.

To further confirm the therapeutic effect of mDexs in mice, immunohistochemical analysis was performed and showed that compared with the saline and AG490 groups, the other groups showed significantly reduced levels of HDV antigens (Fig. 3E). In particular, dyed particles were almost undetectable in the Ub-S-HDAg-Dex group. In line with the serum results, HBsAg did not display a significant difference in liver tissues (Fig. 3F).

Histological changes in liver sections were evaluated in terms of inflammatory cell infiltration and hepatocyte hypertrophy. There were few lymphocytes and more serious hypertrophy in the liver in the saline group, while a larger amount of lymphocyte infiltration was observed in the Ub-S-HDAg-Dex group (Fig. 3G).

### The antiviral effect of Ub-S-HDAg-Dexs was exerted via the JAK/STAT pathway.

**FIG 4:**
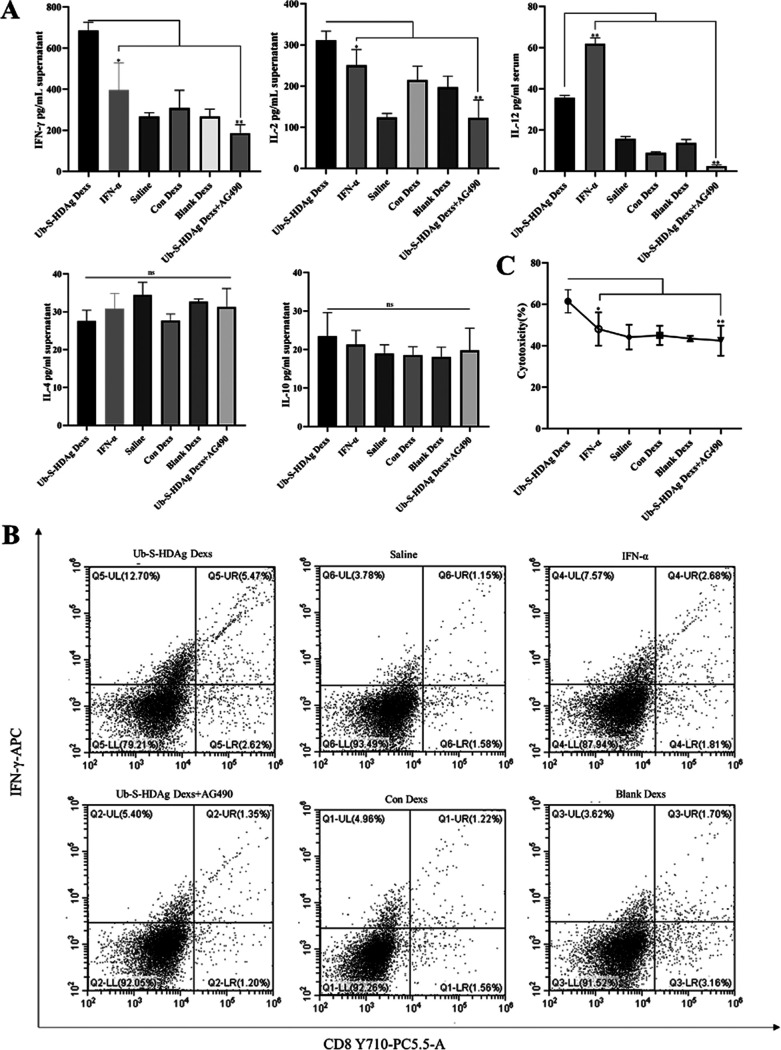
Ub-S-HDAg-Dexs activated a CD8^+^ T cell immune response. (A) Production of the cytokines IFN-γ, IL-2, IL-4, IL-10, and IL-12 in the supernatant after T lymphocyte incubation with S-HDAg for 24 h. (B) The levels of intracellular IFN-γ and CD8^+^ T cells in spleens from immunized HDV replication model mice. (C) Specific CTL activity was assessed using an LDH release assay. P816 was incubated with S-HDAg for 24 h and as the target cocultured with T lymphocytes at an effector:target (E:T) ratio of 10:1 for 24 h. Data are presented as mean ± SD. *n * = 5 animals/condition/experiment. Significance was calculated by one-way ANOVA tests: *, *P* = 0.05; **, *P* = 0.01; ns, nonsignificant.

As shown in Figure 4A, splenocytes from the Ub-S-HDAg-Dex group secreted high levels of IFN-γ and IL-2, while the levels of IL-4 and IL-10 were lower than those in the other groups.

**FIG 5:**
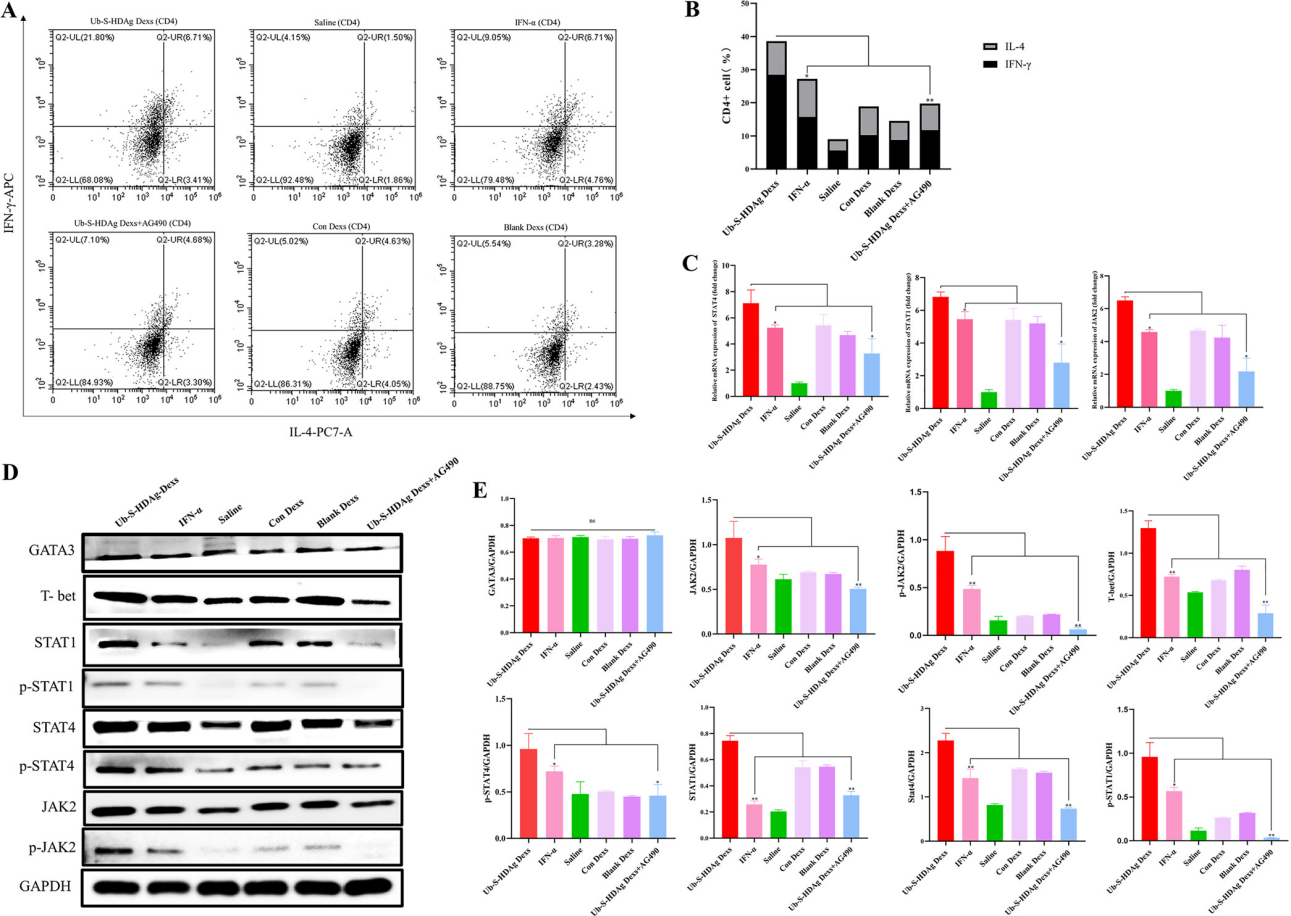
The antiviral effect of Ub-S-HDAg-Dexs was exerted through differentiation into Th1-type cells. At the end of treatment, spleen-derived T lymphocytes were collected. (A and B) CD4^+^ cells were stained with PE-Cy7-conjugated anti-IL-4 and APC-conjugated anti-IFN-γ antibodies and analyzed by flow cytometry. (C) The mRNA expression of JAK2, STAT1, and STAT4. (D and E) Images and quantification of protein expression of T-bet, GATA-3, JAK2, p-JAK2, p-STAT1, STAT1, p-STAT4, and STAT4. Significance was calculated by one-way ANOVA tests: *, *P* = 0.05; **, *P* = 0.01; ns, nonsignificant.

As shown in Fig. 4B and Fig. 5A, B, the percentage of IFN-γ-secreting CD8^+^ and CD4^+^ T cells were higher in the Ub-S-HDAg-Dex group than in the other groups (*P* < 0.01), and Th1 cells were obviously dominant in the Ub-S-HDAg-Dex group.

An lactate dehydrogenase (LDH) release assay was performed to detect HDAg-specific CTL activity against P815/c cells. As shown in Fig. 4C, the percentage of specific cytolysis in the HDAg exosome group was 61.52% at an effector:target (E:T) ratio of 10:1, which was higher than that in the other groups (*P* < 0.01). However, when Ub-S-HDAg-Dexs were administered AG490, the CD8^+^ T cell percentage, Th1/Th2 cell ratio, and released LDH amount were reduced. These results indicated that AG490 weakened the antiviral response.

**FIG 6:**
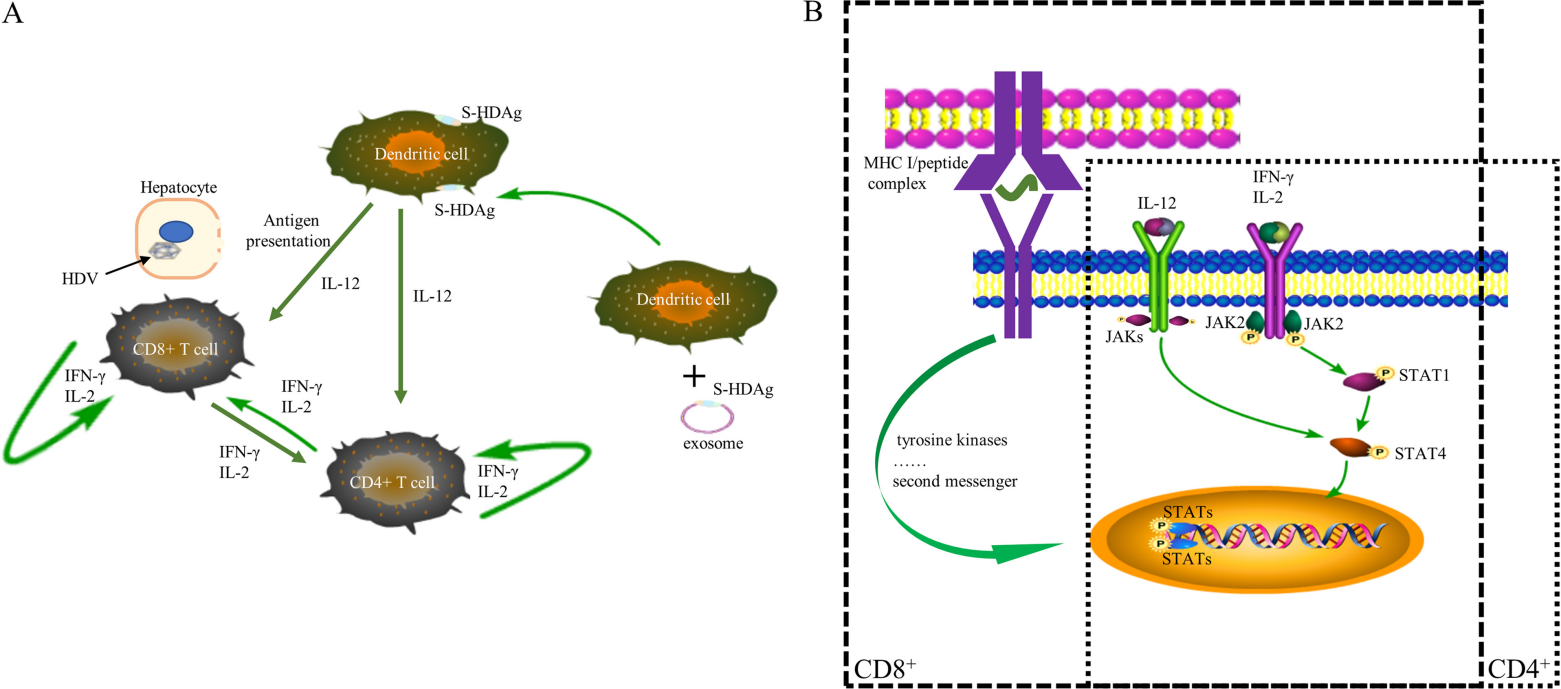
Signal transduction diagram illustrating the immune mechanism of the antiviral effect of Ub-S-HDAg-Dexs. (A) Ub-S-HDAg-Dexs activated DCs to secrete IL-12 and enhance the MHC-I antigen presentation pathway to activate CD8^+^ and CD4^+^ T cells. (B) Ub-S-HDAg-Dexs activated intracellular transcription factors through the MHC-I antigen presentation pathway, increased the levels of IL-2 and IFN-γ, promoted the expression and phosphorylation of STAT1 and STAT4 to induce CD8^+^ T cells, and drove CD4^+^ T cells to differentiate into Th1 cells.

To further confirm the Th1/Th2 cell ratio in HDV replication model mice after treatment, qRT–PCR was performed to analyze the expression of JAK2, STAT1, and STAT4 in T cells (Fig. 5C), and western blotting was performed to analyze the expression of GATA-3, T-bet, p-JAK2, p-STAT1, p-STAT4, JAK2, STAT1, and STAT4. As shown in Fig. 5D and E, the expression of p-JAK2, p-STAT1, p-STAT4, T-bet, STAT1, and STAT4 was significantly upregulated in the Ub-S-HDAg-Dex group compared with IFN-α groups. However, GATA3 expression was not different among the treatment groups. Specifically, the upregulation of T-bet, p-JAK2, p-STAT1, p-STAT4, STAT1, and STAT4 by Ub-S-HDAg-Dexs was reduced by the addition of AG490. However, the protein expression of GATA-3 was not significantly different (Fig. 4C). These findings demonstrated that Ub-S-HDAg-Dexs might regulate the Th1/Th2 cell balance and induce active CD8^+^ T cells to protect against HDV via the JAK/STAT pathway (Fig. 6).

## DISCUSSION

Patients with chronic HBV infection superinfected with HDV have a high risk of rapid progression to liver cirrhosis and hepatocellular carcinoma. Until now, the exact mechanisms that lead to the control of HDV viremia have not been fully defined, and detailed immunological studies are mostly lacking (26–28).

In our study, we found that antigen-modified mDexs contributed to HDV clearance by activating the adaptive immune response via JAK/STAT signaling pathway. Firstly, we constructed HDV replicated mouse model by hydrodynamic transfections. This animal model mimicked some major characteristics of hepatitis D patients that increase in the HDV genome and antigenome in the liver, with a good correlation between the level of intrahepatic replication and that of HDV RNA in the serum (29, 30). Virus-specific CD8^+^ T cells are thought to play a key role in curing HBV/HDV coinfections. However, most studies have found broadly low-level HDV-specific CD4^+^ and CD8^+^ T cell responses in a number of HDV-infected patients, regardless of their disease status (6). Recent studies have demonstrated that a treatment-induced reduction in HDV viremia may facilitate the expansion of HDV-specific T cells *in vitro* (27). Activation of naive CD8^+^ T cells and polarization into effective CTLs requires presentation of MHC I-peptide complexes (signal 1) together with costimulation (signal 2) in the presence of cytokines (signal 3), such as IL-12 and IFN-α (31). IFN-α provides only signal 3 to enhance the CTL response against HDV, while Ub-S-HDAg-Dexs not only provide MHC I-peptide complexes together with costimulatory molecules but may also provide biological components. Proinflammatory cytokines secreted by DCs are enriched in mDexs (9). Consistent with our hypothesis, the results showed Ub-S-HDAg-Dexs had a strong inhibitory effect on HDV replication by enrichment of CTLs response (Fig. 4), resulting in a significant reduction in the viral load. Immunohistochemistry showed that in the Ub-S-HDAg-Dex group, brown-stained particles indicative of HDAg were rarely seen, while increased lymphocyte infiltration was observed in liver tissue (Fig. 3E and G). However, current studies indicate that most HDV RNA in the liver and serum is gone at day 21 in the majority of hydrodynamically transfected mice (24, 30). Mice are not the natural host of HDV, and viral particles eventually stop infecting hepatocytes. In addition, the intact immune system might ultimately eliminate foreign pathogens. These features made it difficult to demonstrate the benefit of our therapy, and the current results might underestimate the treatment efficacy, which could become apparent in a system where replication is persistent in the absence of effective therapy. Nevertheless, AST and ALT levels did not differ among the groups (Fig. 3C and D). Previous data showed that an effective HBV-specific CTL response inhibited viral replication without causing hepatocyte damage but that the aggregation of non-HBV-specific T cells caused pathological liver damage when the HBV-specific CTL response failed to control viral replication (15). Our data indicated that an effective HDV-specific CTL response induced by Ub-S-HDAg-Dexs could ultimately inhibit viral replication without causing hepatocyte damage.

The levels of IFN-γ and IL-2 were almost doubled in Ub-S-HDAg-Dexs group compared with the saline group. Although the levels of IL-4 and IL-10 were not significantly different, IL-4 and IL-10 expression was lower in Ub-S-HDAg-Dex group and higher in the saline group. IL-10 has been found to be abundant in chronic hepatitis B (CHB) patients (32), which indicates that the effect of Th2-type cytokines is stronger than that of Th1-type cytokines. In addition, to complete Th1 differentiation, the Ifng gene was activated while the IL-4 gene was inhibited (33). Cytokines produced by CD4^+^ cells stimulate the activation and differentiation of naive CD8^+^ T cells into effector CTLs. IFN-γ is necessary for the differentiation of Th1 cells, and the activation of Th1 immunity is involved in CTL activity. These demonstrated that benefiting from cytokines secreting from Th1-type cells, the viability of HDV-specific CD8^+^ T cells was further strengthened in the Ub-S-HDAg-Dexs group.

JAK/STAT signaling pathway is crucial in mediating cell differentiation and cytokine production. The relative expression of specific transcription factors modulates the differentiation of Th cells (34). In Th1 cell differentiation, Stat1 and Stat4 are vital (35, 36), and IL-12 stabilizes T-bet and the Th1 cell phenotype via STAT4 (35, 37) (Fig. 6). Our results revealed that the levels of p-JAK2, p-STAT1, p-STAT4, T-bet, STAT1, and STAT4 were significantly upregulated in the Ub-S-HDAg-Dex group. In contrast, the expression of p-JAK2, p-STAT1, p-STAT4, STAT1, and STAT4 was markedly decreased in the Ub-S-HDAg-Dex+AG490 group compared with the Ub-S-HDAg-Dex group, in line with the reduced levels of Th1-type cytokines (IL-2 and IFN-γ). Although the level of IL-12 was higher in the IFN-α group, the level of pathway activation and the antiviral effect in this group were weaker than those in the Ub-S-HDAg-Dex group. P-JAK2, p-STAT1, p-STAT4, STAT1, T-bet, and STAT4 were upregulated in the blank Dex and Con-Dex groups (Fig. 5), in line with the corresponding exosomes derived from mDCs, which have been shown to be immunostimulatory (38). More experiments are required to determine contents of exosomes, which might be beneficial to the adaptive immune response. Thus, taken together, these data indicated that Ub-S-HDAg-Dexs activated the adaptive immune response via JAK/STAT signaling pathway.

In summary, mDexs carrying ubiquitinated HDAg could increase CD8^+^ cell numbers and regulate the Th1/Th2 cell ratio to eliminate HDV in our experiment. Moreover, the antiviral immune response was induced via the JAK/STAT pathway. However, the components of exosomes have not been fully identified and should be further explored. Consequently, treatment with Ub-S-HDAg-Dexs might be a potential therapeutic strategy to eradicate HDV in the future.

## MATERIALS AND METHODS

### Hydrodynamic transfection of a mouse model.

HBV transgenic C57BL/6 mice (female, 6 to 8 weeks old) were purchased from the Southern Mode Company (Shanghai, China). HBsAg and HBV DNA in serum and HBsAg expression in hepatocytes could be detected in the mice. All mice were bred under specific pathogen-free conditions in the Experimental Animal Centre of Shanghai Jiao Tong University Affiliated Sixth People’s Hospital. All experimental procedures were approved by the laboratory animal ethical commissions of Shanghai Jiao Tong University.

Mice were intravenously injected with 25 μg total HDV-encoding plasmid pCMV-HDV-I (+), which was kindly provided by Jeffrey S. Glenn and encoded 1.2-unit-length antigenomes of HDV genotypes, in saline via the tail vein according to a previous method (39). After injection, viremia was confirmed on day 7. The transgenic mice were randomly divided into six groups, with five mice in each group. The mice were injected subcutaneously with 1 μg exosomes (25 μL) in the hind footpads every 3 days for a total of four times and additional intraperitoneal injection of AG490 (500 μg/mouse) into mice in Ub-S-HDAg Dexs+AG490 group. Saline was used as the negative control and was injected subcutaneously in the hind footpads. IFN-α was used as the positive control and administered by intramuscular injection of 1,300 IU/g. Mice were sacrificed at the indicated time points. Splenocytes, livers, and sera were collected for detection.

### Plasmid construction.

The open reading frames (ORFs) of Ub, S-HDAg, and Lamp2 were amplified by PCR, and a fusion gene was designed for cloning into the expression vector pH 149: pLenti-EF1a-EGFP-P2A-Puro-CMV-MCS-3Flag at EcoRI-digested pCMV-C2 by OBiO Technology (Shanghai) Corp., Ltd. The resulting pH 10833: pLenti-EF1a-EGFP-P2A-Puro-CMV-SP-Ub-S-HDAg-Lamp2-3Flag plasmid contained the human Ub-S-HDAg-Lamp2 cDNA sequence under the control of the CMV promoter, and the sequences were detected. To produce lentivirus, these plasmids were transfected into 293T cells together with packaging plasmids.

### Cell culture.

Bone marrow dendritic cells (BMDCs) were collected from the tibias and femurs of C57BL/6 mice (40). After the erythrocytes were lysed, the cells were cultured for 4 h in complete RPMI 1640 medium (HyClone) to remove any nonadherent cells, and the adherent cells were cultured in medium containing 10 ng/mL GM-CSF and 1 ng/mL IL-4 (PeproTech) for 5 days. Then, immature DCs (imDCs) were transfected with pH 10833 (to produce Ub-S-HDAg-Dexs) or pH 149 (as the control plasmid, to produce Con-Dexs) for 48 h and then treated with LPS (1 μg/mL) for 24 h. To produce blank Dexs, imDCs were directly stimulated with LPS for 24 h. The maturation state of DCs was detected by flow cytometric analysis of staining for CD80, CD86, MHC-II, and CD11c (eBioscience). Mature DCs (mDCs) were refed with fresh serum-free medium for another 48 h. Finally, the culture medium was collected for exosome isolation.

### Exosome isolation and identification.

Serum-free conditioned medium was collected at the indicated time points after transfection. Dexs were isolated using a previously described protocol (41). Briefly, the culture supernatant from mDCs was collected and centrifuged at 37°C and 300 × *g* for 10 min. Next, the supernatant was harvested and centrifuged at 4°C and 2,000 × *g* for 20 min. Then, the supernatant was collected again and centrifuged at 10,000 × *g* for 30 min. The supernatant was transferred to 100-kDa MWCO Amicon Ultra15 Centriplus centrifugal ultrafiltration units (Millipore) and centrifuged at 4°C and 1,500 × *g* for 15 min. The residue containing exosomes was washed twice with PBS at 100,000 × *g* and 4°C for 70 min, and finally, the obtained Dexs pellets were resuspended in 100 μL PBS. The protein content of Dexs was quantified with a bicinchoninic acid assay (Solarbio). The ultrastructure and size distribution of exosomes were analyzed by TEM and with a NanoSight instrument (Malvern), respectively. The protein markers Hsp90, TSG101, Alix, and Calnexin (Abcam) were evaluated by immunoblotting. For the uptake studies, purified exosomes were labeled with a PKH26 (red) kit (Sigma) according to previously reported protocols. Briefly, exosomes diluted in PBS were added to 0.5 mL Diluent C. Then, 4 μL PKH26 dye was added to 0.5 mL Diluent C and incubated with the exosome solution for 5 min at room temperature. Two milliliters of PBS were added to prevent excess dye binding, and the mixture was ultracentrifuged to re-extract the exosomes. Exosomes were cocultured with DC2.4 cells, and fluorescence microscopy was used to observe the uptake level at 6 h and 12 h. At 24 h, the cells were washed twice with cold PBS, fixed in 4% paraformaldehyde (Servicebio) for 30 min, and permeabilized with PBS containing 0.1% Triton X-100 for 20 min. The samples were incubated with an anti-Flag antibody (Affinity), followed by incubation with a goat anti-mouse immunoglobulin G secondary antibody (Absin). Subsequently, the cells were stained with DAPI and observed under a fluorescence microscope. Dexs were stored at −80°C for subsequent experiments.

### CTL assay.

T lymphocytes were obtained from splenocytes with nylon wool columns (Wako) in accordance with a previously described protocol (20). P815/c cells (5 × 10^4^ cells/well) seeded in 96-well plates and incubated with antigen for 24 h were used as target cells. T lymphocytes, used as effector cells, were cultured with P815/c cells at a specific E:T ratio (10:1) at 37°C for 4 h. The Cytotoxicity LDH assay kit (Dojindo) was used to assess LDH. The optical density values of the supernatants were recorded at 490 nm. Cytotoxicity (%) was calculated as follows: ([experimental release – effector spontaneous release – target spontaneous release]/[target maximum release – target spontaneous release]) ×100%. Flow cytometry was used to classify T lymphocytes, by staining with PerCP-Cy5.5-conjugated anti-CD8, PE-conjugated anti-CD4, PE-Cy7-conjugated anti-IL-4, and APC-conjugated anti-IFN-γ antibodies.

### Cytokine detection.

Splenocytes (8 × 10^6^ cells/ml) were collected at 19 days after the last treatment and cultured in 6-well plates at 37°C in complete RPMI 1640 culture medium in the presence of 10 μg/mL S-HDAg (Abcam). After 24 h of incubation, the concentrations of IFN-γ, IL-2, IL-4, IL-12, and IL-10 in the supernatants were determined using commercial mouse cytokine ELISA kits (R&D) according to the manufacturer’s protocols. The results are expressed in pg/mL.

### Serological analysis.

Blood collected from the orbital sinus of the mouse was centrifuged at 37°C and 3,000 × *g* for 15 min to obtain serum; blood was collected before the first and after the last treatment. HDV RNA was extracted with the Viral RNA minikit (Baidai) and analyzed using a quantitative PCR kit (Bio-Rad) via the probe method (primers shown in Table S1). The purified plasmid pT7GM, which contains 1,679 bp of the HDV genome, was used as the standard for quantification of HDV RNA by real-time PCR (20). The levels of serum ALT and AST were measured using an ARCHITECT Automatic Biochemistry Analyzer (Abbott Diagnostics).

### Histology and immunohistochemistry.

Liver tissues were fixed in formaldehyde and embedded in paraffin. For histological analysis, deparaffinized sections were stained with hematoxylin and eosin (H&E). For immunohistochemical analysis, deparaffinized sections were treated with 0.3% H_2_O_2_ to inactivate endogenous peroxidases, blocked with 2% goat serum for 30 min at room temperature, and incubated with a goat anti-HBsAg monoclonal antibody (Sigma) or primary human anti-HDAg antibody overnight at 4°C. Following washing with PBS, the sections were stained with a biotinylated secondary antibody (Boster) for 30 min at 37°C followed by a streptavidin-biotin-peroxidase complex for 30 min before being visualized with diaminobenzidine (Boster) and counterstained with hematoxylin.

### Analysis of mRNA and protein expression.

Spleen-derived T lymphocytes were harvested for total RNA isolation with TRIzol Reagent (Invitrogen) following the manufacturer’s instructions. Total RNA was reverse transcribed into cDNA using the PrimeScript RT reagent kit (TaKaRa). Then, quantitative real-time PCR (qRT–PCR) was performed 2×Universal SYBR green Fast qPCR Mix (ABclone) according to previously published protocols (42). GAPDH was used as an internal reference. The PCR primer sequences are listed in Table S1. The PCR conditions were as follows: 95°C for 5 min followed by 40 cycles of 95°C for 5 sec and 60°C for 36 sec. The target gene level was calculated using the 2^−ΔΔCt^ method.

After lysis of harvested T lymphocytes using RIPA lysis buffer containing a protease inhibitor mixture (Beyotime), the protein concentration was determined using a BCA protein assay reagent kit (Solarbio). Protein lysates were separated by 10% SDS-PAGE and then transferred to a PVDF membrane. Rabbit anti-mouse antibodies against GATA-3 (1:1,000; Abcam), Tbx21 (1:1,000; Abcam), STAT1 (1:1,000; Abcam), STAT4 (1:1,000; Abcam), and GAPDH (1:1,000; Abcam) were used as the primary antibodies. HRP-conjugated goat anti-rabbit or anti-mouse immunoglobulin G was used as the secondary antibody. Immunoreactive bands were detected with a Xomat machine (Amersham Pharmacia) after membrane incubation with ECL Selected Western Blotting Detection Reagent (New Cell and Molecular Biotech).

### Statistical analysis.

All data are expressed as the mean ± standard deviation (SD). Differences among groups were compared using one-way ANOVA and post hoc least significant difference tests. Data were analyzed with GraphPad 8.0 software, and *P* < 0.05 was considered statistically significant.
