# Supplementary material for: Ubiquitinated Hepatitis D Antigen-Loaded Microvesicles Induce a Potent Specific Cellular Immune Response to Inhibit HDV Replication in Vivo
Source: Microbiol Spectr. 2021 Dec 15;9(3):e01024-21. doi: 10.1128/Spectrum.01024-21 (PMC8672902; doi:10.1128/Spectrum.01024-21)
Supplement: SUPPLEMENTAL FILE 1 — Supplemental material. Download SPECTRUM01024-21_Supp_1_seq11.pdf, PDF file, 1.1 MB [file spectrum01024-21_supp_1_seq11.pdf]

Figure S1

```
                               1                                                50
               seq      (1)  GGCGTGTACGGTGGGAGGTCTATATAAGCAGAGCTCGTTTAGTGAACCGT
  SP-Ub-S-HDAg-Lamp2    (1)  --------------------------------------------------
                             51                                              100
               seq     (51)  CAGATCGAATTCGCCACCATGTGCCTCTCTCCGGTTAAAGGCGCAAAGCT
  SP-Ub-S-HDAg-Lamp2    (1)  ------------------ATGTGCCTCTCTCCGGTTAAAGGCGCAAAGCT
                             101                                             150
               seq    (101)  CATCCTGATCTTTCTGTTCCTAGGAGCCGTTCAGTCCAATGCAATGCAGA
  SP-Ub-S-HDAg-Lamp2   (33)  CATCCTGATCTTTCTGTTCCTAGGAGCCGTTCAGTCCAATGCAATGCAGA
                             151                                             200
               seq    (151)  TCTTCGTGAAGACCCTGACTGGCAAGACCATCACCCTGGAGGTGGAGCCC
  SP-Ub-S-HDAg-Lamp2   (83)  TCTTCGTGAAGACCCTGACTGGCAAGACCATCACCCTGGAGGTGGAGCCC
                             201                                             250
               seq    (201)  AGTGACACCATCGAGAACGTGAAGGCCAAGATCCAGGATAAAGAGGGCAT
  SP-Ub-S-HDAg-Lamp2  (133)  AGTGACACCATCGAGAACGTGAAGGCCAAGATCCAGGATAAAGAGGGCAT
                             251                                             300
               seq    (251)  CCCCCCTGACCAGCAGAGGCTGATCTTTGCCGGCAAGCAGCTGGAAGATG
  SP-Ub-S-HDAg-Lamp2  (183)  CCCCCCTGACCAGCAGAGGCTGATCTTTGCCGGCAAGCAGCTGGAAGATG
                             301                                             350
               seq    (301)  GCCGCACCCTCTCTGATTACAACATCCAGAAGGAGTCAACCCTGCACCTG
  SP-Ub-S-HDAg-Lamp2  (233)  GCCGCACCCTCTCTGATTACAACATCCAGAAGGAGTCAACCCTGCACCTG
                             351                                             400
               seq    (351)  GTCCTTCGCCTGAGAGGTGCTAGAAGCCGGTCCGAGTCGAGGAAGAACCG
  SP-Ub-S-HDAg-Lamp2  (283)  GTCCTTCGCCTGAGAGGTGCTAGAAGCCGGTCCGAGTCGAGGAAGAACCG
                             401                                             450
               seq    (401)  CGGAGGGAGAGAAGAGATCCTCGAGCAGTGGGTGGCCGGAAGAAAGAAGT
  SP-Ub-S-HDAg-Lamp2  (333)  CGGAGGGAGAGAAGAGATCCTCGAGCAGTGGGTGGCCGGAAGAAAGAAGT
                             451                                             500
               seq    (451)  TAGAGGAACTCGAGAGAGACCTCCGGAAGACAAAGAAGAAACTCAAAAAG
  SP-Ub-S-HDAg-Lamp2  (383)  TAGAGGAACTCGAGAGAGACCTCCGGAAGACAAAGAAGAAACTCAAAAAG
                             501                                             550
               seq    (501)  ATTGAGGACGAAAATCCCTGGCTGGGGAACATCAAAGGAATTCTCGGAAA
  SP-Ub-S-HDAg-Lamp2  (433)  ATTGAGGACGAAAATCCCTGGCTGGGGAACATCAAAGGAATTCTCGGAAA
                             551                                             600
               seq    (551)  GAAGGATAAGGATGGAGAGGGGGCTCCCCCGGCGAAGAGGGCCCGAACGG
  SP-Ub-S-HDAg-Lamp2  (483)  GAAGGATAAGGATGGAGAGGGGGCTCCCCCGGCGAAGAGGGCCCGAACGG
                             601                                             650
               seq    (601)  ACCAGATGGAGGTAGACTCCGGACCTAGGAAGAGGCCTCTCAGGGGAGGA
  SP-Ub-S-HDAg-Lamp2  (533)  ACCAGATGGAGGTAGACTCCGGACCTAGGAAGAGGCCTCTCAGGGGAGGA
                             651                                             700
               seq    (651)  TTCACCGACAAGGAGAGGCAGGATCACCGACGAAGGAAGGCCCTCGAGAA
  SP-Ub-S-HDAg-Lamp2  (583)  TTCACCGACAAGGAGAGGCAGGATCACCGACGAAGGAAGGCCCTCGAGAA
                             701                                             750
               seq    (701)  CAAGAAGAAGCAGCTATCGGCGGGAGGCAAGAACCTCAGCAAGGAGGAAG
  SP-Ub-S-HDAg-Lamp2  (633)  CAAGAAGAAGCAGCTATCGGCGGGAGGCAAGAACCTCAGCAAGGAGGAAG
                             751                                             800
               seq    (751)  AAGAGGAACTCAGGAGGTTGACCGAGGAAGACGAGAGAAGGGAAAGAAGA
  SP-Ub-S-HDAg-Lamp2  (683)  AAGAGGAACTCAGGAGGTTGACCGAGGAAGACGAGAGAAGGGAAAGAAGA
                             801                                             850
               seq    (801)  GTAGCCGGCCCGCCGGTTGGGGGTGTGATCCCCCTCGAAGGTGGATCGAG
  SP-Ub-S-HDAg-Lamp2  (733)  GTAGCCGGCCCGCCGGTTGGGGGTGTGATCCCCCTCGAAGGTGGATCGAG
                             851                                             900
               seq    (851)  GGGAGCGCCCGGGGGCGGCTTCGTCCCCAGTCTGCAGGGAGTCCCGGAGT
  SP-Ub-S-HDAg-Lamp2  (783)  GGGAGCGCCCGGGGGCGGCTTCGTCCCCAGTCTGCAGGGAGTCCCGGAGT
                             901                                             950
               seq    (901)  CCCCCTTCTCTCGGACCGGGGAGGGGCTGGACATCAGGGGAAACCGGGGA
  SP-Ub-S-HDAg-Lamp2  (833)  CCCCCTTCTCTCGGACCGGGGAGGGGCTGGACATCAGGGGAAACCGGGGA
                             951                                            1000
               seq    (951)  TTTCCATTGATAGTTAATTTGACAGATTCAAAGGGTACTTGCCTTTATGC
  SP-Ub-S-HDAg-Lamp2  (883)  TTTCCATTGATAGTTAATTTGACAGATTCAAAGGGTACTTGCCTTTATGC
                             1001                                           1050
               seq   (1001)  AGAATGGGAGATGAATTTCACAATAACATATGAAACTACAAACCAAACCA
  SP-Ub-S-HDAg-Lamp2  (933)  AGAATGGGAGATGAATTTCACAATAACATATGAAACTACAAACCAAACCA
                             1051                                           1100
               seq   (1051)  ATAAAACTATAACCATTGCAGTACCTGACAAGGCGACACACGATGGAAGC
  SP-Ub-S-HDAg-Lamp2  (983)  ATAAAACTATAACCATTGCAGTACCTGACAAGGCGACACACGATGGAAGC
```

```
                                 1101                                              1150
                 seq    (1101)   AGTTGTGGGGATGACCGGAATAGTGCCAAAATAATGATACAATTTGGATT
SP-Ub-S-HDAg-Lamp2    (1033)   AGTTGTGGGGATGACCGGAATAGTGCCAAAATAATGATACAATTTGGATT
                                 1151                                              1200
                 seq    (1151)   CGCTGTCTCTTGGGCTGTGAATTTTACCAAGGAAGCATCTCATTATTCAA
SP-Ub-S-HDAg-Lamp2    (1083)   CGCTGTCTCTTGGGCTGTGAATTTTACCAAGGAAGCATCTCATTATTCAA
                                 1201                                              1250
                 seq    (1201)   TTCATGACATCGTGCTTTCCTACAACACTAGTGATAGCACAGTATTTCCT
SP-Ub-S-HDAg-Lamp2    (1133)   TTCATGACATCGTGCTTTCCTACAACACTAGTGATAGCACAGTATTTCCT
                                 1251                                              1300
                 seq    (1251)   GGTGCTGTAGCTAAAGGAGTTCATACTGTTAAAAATCCTGAGAATTTCAA
SP-Ub-S-HDAg-Lamp2    (1183)   GGTGCTGTAGCTAAAGGAGTTCATACTGTTAAAAATCCTGAGAATTTCAA
                                 1301                                              1350
                 seq    (1301)   AGTTCCATTGGATGTCATCTTTAAGTGCAATAGTGTTTTAACTTACAACC
SP-Ub-S-HDAg-Lamp2    (1233)   AGTTCCATTGGATGTCATCTTTAAGTGCAATAGTGTTTTAACTTACAACC
                                 1351                                              1400
                 seq    (1351)   TGACTCCTGTCGTTCAGAAATATTGGGGTATTCACCTGCAAGCTTTTGTC
SP-Ub-S-HDAg-Lamp2    (1283)   TGACTCCTGTCGTTCAGAAATATTGGGGTATTCACCTGCAAGCTTTTGTC
                                 1401                                              1450
                 seq    (1401)   CAAAATGGTACAGTGAGTAAAAATGAACAAGTGTGTGAAGAAGACCAAAC
SP-Ub-S-HDAg-Lamp2    (1333)   CAAAATGGTACAGTGAGTAAAAATGAACAAGTGTGTGAAGAAGACCAAAC
                                 1451                                              1500
                 seq    (1451)   TCCCACCACTGTGGCACCCATCATTCACACCACTGCCCCGTCGACTACAA
SP-Ub-S-HDAg-Lamp2    (1383)   TCCCACCACTGTGGCACCCATCATTCACACCACTGCCCCGTCGACTACAA
                                 1501                                              1550
                 seq    (1501)   CTACACTCACTCCAACTTCAACACCCACTCCAACTCCAACTCCAACTCCA
SP-Ub-S-HDAg-Lamp2    (1433)   CTACACTCACTCCAACTTCAACACCCACTCCAACTCCAACTCCAACTCCA
                                 1551                                              1600
                 seq    (1551)   ACCGTTGGAAACTACAGCATTAGAAATGGCAATACTACCTGTCTGCTGGC
SP-Ub-S-HDAg-Lamp2    (1483)   ACCGTTGGAAACTACAGCATTAGAAATGGCAATACTACCTGTCTGCTGGC
                                 1601                                              1650
                 seq    (1601)   TACCATGGGGCTGCAGCTGAACATCACTGAGGAGAAGGTGCCTTTCATTT
SP-Ub-S-HDAg-Lamp2    (1533)   TACCATGGGGCTGCAGCTGAACATCACTGAGGAGAAGGTGCCTTTCATTT
                                 1651                                              1700
                 seq    (1651)   TTAACATCAACCCTGCCACAACCAACTTCACCGGCAGCTGTCAACCTCAA
SP-Ub-S-HDAg-Lamp2    (1583)   TTAACATCAACCCTGCCACAACCAACTTCACCGGCAGCTGTCAACCTCAA
                                 1701                                              1750
                 seq    (1701)   AGTGCTCAACTTAGGCTGAACAACAGCCAAATTAAGTATCTTGACTTTAT
SP-Ub-S-HDAg-Lamp2    (1633)   AGTGCTCAACTTAGGCTGAACAACAGCCAAATTAAGTATCTTGACTTTAT
                                 1751                                              1800
                 seq    (1751)   CTTTGCTGTGAAAAATGAAAAACGGTTCTATCTGAAGGAAGTGAATGTCT
SP-Ub-S-HDAg-Lamp2    (1683)   CTTTGCTGTGAAAAATGAAAAACGGTTCTATCTGAAGGAAGTGAATGTCT
                                 1801                                              1850
                 seq    (1801)   ACATGTATTTGGCTAATGGCTCAGCTTTCAACATTTCCAACAAGAACCTT
SP-Ub-S-HDAg-Lamp2    (1733)   ACATGTATTTGGCTAATGGCTCAGCTTTCAACATTTCCAACAAGAACCTT
                                 1851                                              1900
                 seq    (1851)   AGCTTCTGGGATGCCCCTCTGGGAAGTTCTTATATGTGCAACAAAGAGCA
SP-Ub-S-HDAg-Lamp2    (1783)   AGCTTCTGGGATGCCCCTCTGGGAAGTTCTTATATGTGCAACAAAGAGCA
                                 1901                                              1950
                 seq    (1901)   GGTGCTTTCTGTGTCTAGAGCGTTTCAGATCAACACCTTTAACCTAAAGG
SP-Ub-S-HDAg-Lamp2    (1833)   GGTGCTTTCTGTGTCTAGAGCGTTTCAGATCAACACCTTTAACCTAAAGG
                                 1951                                              2000
                 seq    (1951)   TGCAACCTTTTAATGTGACAAAAGGACAGTATTCTACAGCTCAAGACTGC
SP-Ub-S-HDAg-Lamp2    (1883)   TGCAACCTTTTAATGTGACAAAAGGACAGTATTCTACAGCTCAAGACTGC
                                 2001                                              2050
                 seq    (2001)   AGTGCAGATGAAGACAACTTCCTTGTGCCCATAGCGGTGGGAGCAGCTCT
SP-Ub-S-HDAg-Lamp2    (1933)   AGTGCAGATGAAGACAACTTCCTTGTGCCCATAGCGGTGGGAGCAGCTCT
                                 2051                                              2100
                 seq    (2051)   GGGAGGAGTACTTATTCTAGTGTTGCTGGCTTATTTTATTGGTCTCAAGC
SP-Ub-S-HDAg-Lamp2    (1983)   GGGAGGAGTACTTATTCTAGTGTTGCTGGCTTATTTTATTGGTCTCAAGC
                                 2101                                              2150
                 seq    (2101)   GCCATCATACTGGATATGAGCAATTTGAATTCGACTACAAGGATGACGAT
SP-Ub-S-HDAg-Lamp2    (2033)   GCCATCATACTGGATATGAGCAATTT------------------------
```

Figure S1: Comparison between actual sequencing results and predicted sequencing results. Seq from the plasmid we constructed was as same as the sequencing SP-Ub-S-HDAg-Lamp2 predicted.

Figure S2

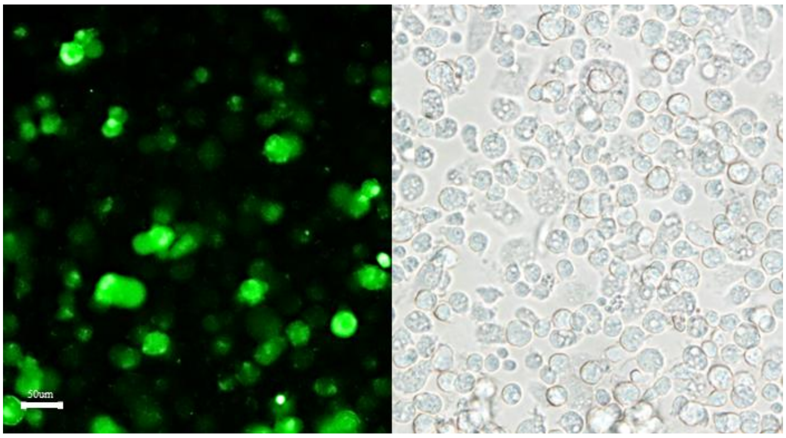

Figure S2: imDCs transfected with the Ub-S-HDAg-lamp2b plasmid showed green fluorescence.

Figure S3

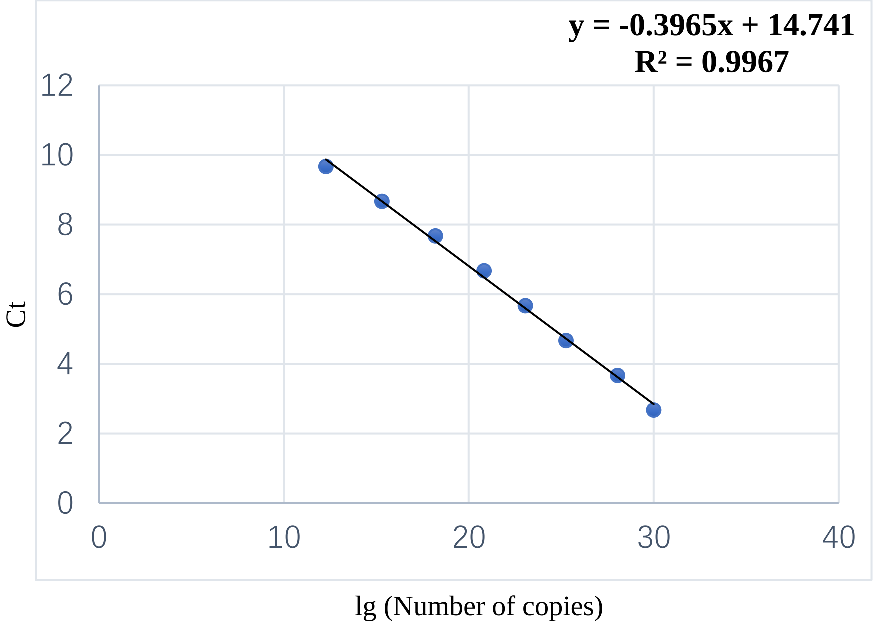

Figure S3: Standard curve line of HDV. The standard purified plasmid pT7GM of HDV was used to draw the standard curve line by real-time PCR.

Table S1. Oligonucleotide sequences used for Q-PCR analysis.

Primer	Sequences (5'-3')	Fragment length (bp)	Annealing temperature (˚C)
HDV	F 5’-GGCWCTCCCTTAGCCATCCG-3’<br>R 5’-GGTCGGCATGGCATCTCCA-3’<br>Probe: 5’-CTCCTWCGGATGCCCAGGTCGGAC-3’	120	60
JAK1	F 5’-CAGATGCCCACCATTACC-3’<br>R 5’-CCCTCTTCACTCCCTTCC-3’	133	56
JAK2	F 5’-GGCAGCAGCAGAACCTAC-3’<br>R 5’-ATGTAACACCGCCATCCC-3’	177	58
JAK3	F 5’- CCTGCCTGTTTATCATTCGCT -3’<br>R 5’- AAGACTTGAGTGTCCACGTCC -	105	60
TYK2	F 5’-GGGTCACCTTCAGCCAGACA-3’<br>R 5’-GACCTTAGCCTGTGCATTGTAGAGT-3’	122	62
STAT1	R 5’-TGGTGAAATTGCAAGAGCTG-3’<br>F 5’-TGTGTGCGTACCCAAGATGT-3’	119	60
STAT4	F 5’-CCTGCTGTTGGTTGGTGT-3’<br>R 5’-CTTGAGGCTTTCCTGTGC-3’	266	56
GAPDH	F 5’-AGGTCGGTGTGAACGGATTTG-3’<br>R 5’-GGGGTCGTTGATGGCAACA-3’	95	60
Tbx21	F 5’- CTAAAGCTCACCAACAACAAGGG-3’<br>R 5’- CAGATGCGTACATGGACTCAAAG-3’	280	60
GATA-3	F 5’- TACAGCTCTGGACTCTTCCCAC-3’<br>R 5’- TTCGCTTGGGCTTGATAAGG-3’	235	60
